# Health Technology Assessment of CEM Pulpotomy in Permanent Molars with Irreversible Pulpitis

**Published:** 2013-12-24

**Authors:** Shahram Yazdani, Mohammad-Pooyan Jadidfard, Bahareh Tahani, Ali Kazemian, Omid Dianat, Laleh Alim Marvasti

**Affiliations:** a *Educational Development Center (EDC), Shahid Beheshti University of Medical Sciences, Tehran, Iran; *; b * Department of Community Oral Health, School of Dentistry, Shahid Beheshti University of Medical Sciences, Tehran, Iran; *; c * Department of Oral Public Health, Dental School, Isfahan University of Medical Science, Isfahan, Iran; *; d * Department of Endodontics, Dental School, Shahid Beheshti University of Medical Sciences, Tehran, Iran; *; e *Dental Research Center, Research Institute of Dental Sciences, Shahid Beheshti University of Medical Sciences, Tehran, Iran;*; f * Iranian Center for Endodontic Research, Research Institute of Dental Sciences, Shahid Beheshti University of Medical Sciences, Tehran, Iran*

**Keywords:** Calcium Enriched Mixture, CEM Cement, Endodontics, Health Technology, Pulpitis, Pulpotomy, Root Canal Therapy, Vital Pulp Therapy

## Abstract

**Introduction**: Teeth with *irreversible *pulpitis usually undergo root canal therapy (RCT). This treatment modality is often considered disadvantageous as it removes vital pulp tissue and weakens the tooth structure. A relatively new concept has risen which suggests vital pulp therapy (VPT) for irreversible pulpitis. VPT with calcium enriched mixture (VPT/CEM) has demonstrated favorable treatment outcomes when treating permanent molars with irreversible pulpitis. This study aims to compare patient related factors, safety and organizational consideration as parts of health technology assessment (HTA) of the new VPT/CEM biotechnology when compared with RCT.** Materials and Methods:** Patient related factors were assessed by looking at short- and long-term clinical success; safety related factors were evaluated by a specialist committee and discussion board involved in formulating healthcare policies. Organizational evaluation was performed and the social implications were assessed by estimating the costs, availability, accessibility and acceptability. The impact of VPT/CEM biotechnology was assessed by investigating the incidence of irreversible pulpitis and the effect of this treatment on reducing the burden of disease.** Results:** VPT/CEM biotechnology was deemed feasible and acceptable like RCT; however, it was more successful, accessible, affordable, available and also safer than RCT. **Conclusion:** When considering socioeconomic implications on oral health status and oral health-related quality of life of VPT/CEM, the novel biotechnology can be more effective and more efficient than RCT in mature permanent molars with irreversible pulpitis.

## Introduction

Oral health can significantly affect quality of life [1]. Dental caries is one of the most common chronic diseases of adults and children in both developed and developing nations, even though the etiology and prevention of this disease is well known. Caries can affect growth and well being in young children [2], and is the most common cause of head and neck pain in humans [3].

Patients, especially from deprived backgrounds, often avoid dental treatment as the costs incurred can be severe [4], and the only alternative to root canal therapy (RCT) may be tooth extraction [5]. According to current evidence based protocols, RCT is indicated once the pulp is diagnosed with irreversible pulpitis and in teeth with necrotic pulps [6]. The fair prognosis of RCT (68-85%) is documented [7]. Besides being rather expensive, complicated, time-consuming, and non-conservative, RCT has the capacity of weakening the tooth structure and thus can initiate a restorative spiral [8]. Moreover, it is often considered a difficult procedure for general dentists.

Although irreversible pulpitis is often associated with spontaneous or prolonged pain after a specific trigger, it can sometimes present without any symptoms [9]. In other words, correlation of clinical signs and symptoms with histopathological status of the pulp is not always accurate. 

Ideally, treatment of carious vital teeth should aim to alleviate pain, remove affected/infected tissue, conserve pulp vitality and finally restore function and aesthetics. Previous reports have indicated that vital pulp therapy (VPT) can only be carried out on *reversibly* inflamed pulps of mature teeth that are traumatically or iatrogenically exposed [10], or in cariously exposed immature teeth [11]. However, some schools of thought have advocated the use of VPT on mature permanent teeth diagnosed with *irreversible* pulpitis [12-15]. During VPT, the infected dentin, bacteria/their byproduct and also the affected pulp tissue are removed. The remaining pulp is sealed with a biocompatible material, and so pulp vitality is maintained. Pulpotomy has been successful in immature teeth with traumatically or cariously exposed pulps [16, 17]. However, the use of this treatment in mature permanent teeth has been controversial [11]. The one- and two-year extensive multi-centered non-inferiority randomized clinical trials that have been assessed here, have shown radiographic and clinical success with VPT using a bio-regenerative endodontic material, calcium enriched mixture cement (VPT/CEM) [18, 19]. The size of the study and results attained in the clinical trials are more than adequate for assessing effectiveness and efficacy of this novel bio-technology [20]. Therefore, assessment of this new biotechnology may generally prove to be invaluable for oral healthcare and maintaining pulp vitality. 

There are ~6000 government funded primary dental care centers in Iran which do not even provide RCT for their patients; moreover, not all general dentist (GD) can perform RCT to acceptable standards [21]. In this health technology assessment (HTA), we aimed to compare VPT/CEM versus RCT in teeth diagnosed with irreversible pulpitis. 

## Material and Methods

Based on the established format for reporting HTA, this study assessed the implementation of VPT/CEM technology from different aspects such as effectiveness, safety (possible risks), organizational and social implications [22-24].

*Phase I* was patient based, where clinical success and reduction/elimination of sign/symptoms as well as level of evidence of the clinical trial was assessed; in *Phase II* safety and the risks of the technology were evaluated; and finally in *Phase III* socio-economic (*i.e.* impact/need, availability and applicability/feasibility) and dental health policy making were addressed. Moreover, a critical appraisal was made of intervention and clinical results to assess the organizational and social implications.

**Setting and participants**

This health technology assessment intends to evaluate a multicenter randomized clinical trial (with non-inferiority design) that was conducted in Iran. This study has six-, twelve- and twenty four-month results; therefore the results analyzed for HTA would provide a high level of evidence. Details of the trial can be found in the previously published articles [18, 19, 25]. Briefly, participants in the trial were patients presenting to 23 University dental clinics throughout 4 states, diagnosed with caries extending to the pulp and *irreversible pulpitis* in mature molars after radiographic and clinical examinations. Considering that this was a national project, the patients can be deemed a mixed sample.

***Intervention***

In the RCT group, the treatment was conducted using step-back technique. To assess the novel health biotechnology, VPT/CEM was the test group; treatment was carried out as described previously [18].

**Phase I: Main outcome measure**

Evaluation of the efficacy of a clinical trial is usually based on the subjective and objective short- and intermediate-term outcome measures; for example the patients’ signs and symptoms, experimental results or the absence/presence of disease. The long-term outcome/prognosis are based on patients’ health and wellbeing; consequently quality of life, functionality, and death are less likely to be evaluated in this type of study [26]. It can be argued that tooth retention will indeed improve the quality of life and functionality of the individual. The efficacy of the VPT/CEM new biotechnology is based on reduction or elimination of pain (short-term) and maintaining the tooth vitality and functionality (intermediate/long-term outcome).

Pain is one of the most common reasons for patients to recourse to the dental clinics, especially with so called *irreversible* pulpitis. Intervention with pulpectomy and if time permits, RCT, results in reduction or suspension of pain. In one study, pain outcome measures were assessed preoperatively as well as 6, 12, 36, 48, and 60 h and 3 to 7 days postoperatively using quantified numerical pain rating scale (NRS) [25]. Based on the reported pain scale, the postoperative continuity of moderate to severe pain in h, the degree of pain reduction in the first 24 h, the time taken to reach pain free status and the number of patients that had no pain, were recorded. Time taken to reach pain free status for the two groups was analyzed using the Kaplan-Meier Test.

Clinical and radiographic evaluations were conducted at regular intervals as described in previous publications, and the clinical subjective symptoms and objective observation of inflammation and/or infections were made [18, 19]. Objective signs including abscess, swelling, sinus tract, redness, and tenderness were examined. Radiographic assessment was made and the results were divided into the following categories; teeth with normal contour and width of periodontal ligament (PDL) were termed *“healed”*, teeth with a clearly decreased size of the periapical radiolucency were judged as *“healing”*, and teeth with unchanged, increased, or new periapical radiolucency were categorized as *“failed”.*

The reported non-inferiority clinical trials were assessed using the standardized CONSORT check list [18, 19, 25]. The levels of evidence (LoE, Oxford), and therefore clinical and social significance was also assessed.

**Phase II: Safety measures**

Most HTA studies have been based on medical biotechnology rather than dental technology. Therefore, to accurately verify and apply these techniques, expert opinion was elicited where formal evidence was not available. A committee and discussion board was established with representatives from Center of Clinical Excellence (CCE), experts in the field of HTA, the researchers, medical professionals involved in formulating healthcare policies, and endodontists with no material/scientific interest in the research. They were requested to discuss and assess the safety margins of this new endodontic biotechnology in vital dental tissue based on published evidence and expert opinion. Ultimately, they were to reach definitive conclusions through focused group discussions.

**Phase III: Organizational measures**

Several measurements were considered for assessing the organizational aspects of the HTA including access, availability and cost. Organizational and professional implications can be addressed with system-related outcomes, such as required personnel. Organizational measures (*e.g.* access) affect the feasibility of implementation of a new technology and the degree of effectiveness following implementation; i.e. human resource or educational prerequisites must be available and accessible. These measures were defined in the same committee and discussion board that assessed the safety margins. According to their consensus, the proposed endodontic biotechnology which aims to attain non-inferiority/equivalency and ultimately superiority, should overcome the limitations of RCT in the fields below.

**Access**

Different aspects should be considered to assure that a technology is accessible; prerequisites for its implementation should be available and the cost barriers should be considered.

***a) Availability of technique***

For this clinical trial, both RCT and pulpotomy had to be available treatments. That is, financial support for expendable and non expendable goods, trained dental professionals, and the right equipment (radiographic units, dental units, dental equipment and materials) had to be available for smooth running of this treatment method especially in deprived rural regions of Iran. The availability of the new biotechnology was then to be compared to RCT within the centers.

***b) Costs***

*Direct costs;* root canal treatment is believed to be a lengthy and costly procedure, which is not supported by the public insurance in Iran. Therefore, in deprived regions of Iran even if the patient provides informed consent, the equipment and materials for RCT is available (which is not in most cases), and dental professionals are adequately trained, the only feasible option would then be extraction of the involved tooth because of the patients’ not being able to afford RCT.* Indirect costs* of CEM/VPT were also compared to RCT. The number of visits to the dental clinic for both treatment groups was considered.

**Impact**

To assess the impact of this new biotechnology in the community at large, the percentage of patients requiring this type of treatment need to be recorded. A literature review conducted by the research team was not able to find studies (conducted in Iran) which report the incidence of teeth with established irreversible pulpitis requiring root canal treatment. Therefore a cross sectional descriptive study, to elicit burden of irreversible pulpitis among other classifications of pulpitis, was performed. Studies that determine the incidence of this disease require a specific number of samples and as there were no previous data, the expected prevalence was taken to be 50% to give the greatest sample requirement of 385 subjects.

Dental records of patients from Endodontic Department of Shahid Beheshti Dental School were selected to form the samples of this study. Patient records from this department were selected on a randomized basis, as all patients with dental pain and endodontic problems were initially referred there and then redistributed among undergraduates or postgraduate students. The patient records, radiographs and OPG which were compiled by dental students (under the supervision of an endodontist) and were analyzed by an endodontist and dental public health PhD student (dentist). Inter-examiner agreement regarding the diagnoses was confirmed. Records with missing radiographs, vague results or inter-examiner disagreement were disregarded; these were then replaced with other records.

The tooth number and type as well as the pulp and periapical status were recorded for each sample. The pulpal status was divided into four categories: *normal, reversible pulpitis, irreversible pulpitis,* and *necrosis*. Periapical status was also subdivided into: *normal, acute apical periodontitis (*AAP*), chronic apical periodontitis (*CAP*), acute apical abscess, chronic apical abscess* or *condensing osteitis*.

## Results

**Phase I: Main outcome measure**

Using the CONSORT check list, results were internally validated and deemed reliable; in addition, the randomized clinical trial was graded as *Level of Evidence *1 (LoE 1) and significance of these results was evident.

Based on the reported randomized clinical trial, there was no significant difference in preoperative pain levels experienced between the two patient groups (VPT/CEM and RCT) [25]. During the first 7 postoperative days, patients in VPT/CEM group experienced less pain compared with the RCT group (*P*>0.001). The greatest reduction in pain occurred in the first 24 h in both groups; however, number of patients reaching pain free status in the VPT/CEM group was greater than the RCT group (73% *vs.* 48%, *P*<0.05).

***Clinical success rates***

Clinical success was based on the absence of signs and symptoms of inflammation/infection as well as tenderness to percussion. There was no significant difference between the two groups at 6-month, 1- and 2-year follow-ups.

***Radiographic success rates***

Compared to RCT, the new biotechnology, VPT/CEM, demonstrated greater success rates after one and two years (1-year: 92.2% *vs.* 70.3%, *P*=0.001; 2-years: 86.7% *vs.* 79.5%, *P*=0.053). As time passed the success rates became more favorable in RCT group. While in CEM/VPT group the presence of preoperative radiographic periapical lesion did not significantly affect the success rate, in RCT group it did [18].

***Sensitivity analyses***

To assess the sensitivity of radiographic success rate, *worse case analysis* was used; that is, teeth which underwent VPT/CEM but had uncertain status after one year were added to the VPT failure subgroup, whereas , in RCT group, the uncertain subgroup were added to the successful cases. This did not alter the results of the chi-square statistical analyses [18].

***Quality of treatment***

The quality of treatment based on radiographic findings and in accordance to modified Strindberg Criteria was significantly different between the two groups (*P*<0.001) [27]; with 92.8% of VPT/CEM and only 66.3% of RCT cases having achieved good quality treatment. Moreover there was significant relationship between the quality of treatment and one-year post operative radiographic success rates [18].

**Phase II: Safety measure**

The group agreed that VPT/CEM for teeth with irreversible pulpitis need to meet six criteria. It was able to get 5 positive and 1 negative responses at this stage of analyses.

***a) Iatrogenic errors occurring during treatment (Positive)***

Compared to RCT, VPT/CEM was considered a much simpler treatment and therefore likely to have less complications and procedural errors such as broken instrument, ledge formation, canal transport/perforations or hypochlorite accident. RCT has many more hazardous steps increasing the risk of serious errors.

***b) Pain experience (Positive)***

Again VPT/CEM was assumed to significantly reduce post operative pain. In addition, the amount of painkillers taken after VPT/CEM was significantly less than the RCT group (*P*>0.001).

***c) Radiographic requirement and x-ray radiation (Positive)***

This item was considered to be lower for VPT/CEM compared to RCT; as RCT usually requires working and postoperative radiographs. Not only VPT/CEM will reduce radiation dose for the patient and thus increases his/her safety, but also may significantly reduce treatment costs.

***d) Longevity of Tooth (Positive)***

RCT increases the risk of tooth fracture due to removal of significant amounts of tooth structure. RCT can initiate a restorative spiral which overtime might result in tooth loss and therefore a decrease in functionality and quality of life, [28]. During VPT/CEM treatment a greater amount of tooth structure is preserved and therefore the risk of tooth fracture is considerably reduced.

***e)*** ***Biocompatibility and non-carcinogenicity of materials (Positive)***

The committee pointed to previous studies which showed that CEM cement is non-toxic and biocompatible which can allow tissue regeneration [29, 30].

***f) Ability to retreat in cases of failure (Negative)***

General opinion was negative for this safety variable. Short- or immediate term failure may occur due to two reasons: 1) *incorrect case selection *and 2) *failure of dentine bridge formation at the canal orifice* [31]. Whereas dentine bridge formation does assume short-term success, in the long-term if the VPT eventually fails and retreatment is required, the canals would be blocked with a dentine bridge. Thus retreatment may be difficult, if not impossible. The other treatment options would be surgical endodontics (a high cost treatment option requiring specific skills) or extraction. A few members of the panel thought that access to the root canals was still achievable as root canal obliteration had not been seen beneath the canal orifices; this was further supported by the two-year follow-ups of the study [19].

**Phase III: Organizational measures**

***Accessibility***

*Availability;* this new biotechnology can be used both in private practice and primary clinics as it does not require any extra specialist skills (unlike molar RCT) or materials, except for CEM cement which is readily available in Iran. *American Dental Association* (ADA) has categorized RCT of molars, prosthesis, orthodontics and surgery as treatments requiring high levels of skill. On the other hand, CEM cement pulpotomy does not require negotiation and preparation of canals, obturation, and *etc*., therefore is simpler to perform. Extra equipment is not also needed for this technique unlike RCT, which may require operative microscope, radiography unit, apex locator, rotary instrument/motor, and so on. Also the diagnoses and treatment plan may be carried out without radiographs just based on sign and symptoms in deprived regions where radiographic equipment is not available.

*Feasibility;* the above mentioned considerations and the fact that general dentists could be easily trained (workshops were nationally available as a 4-h course) made the treatment more feasible than RCT. Radiographs are also not compulsory for CEM/VPT and therefore treatment can be carried out with basic dental amenities. Worldwide epidemiological research shows that an average of 30-65% of RCTs that were carried out by general dentists, failed [21].

**Costs**

*Direct cost:* immediate chair side cost for patients was recently reported; CEM/VPT had significantly lower cost in Iran, *i.e.* 44.5 k per molar tooth compared to 171.5 k for RCT [19].

*Indirect cost:* the general consensus of the team was that VPT/CEM involved less equipment, materials, travel costs, specialist centers and radiography; therefore would be less expensive. It was thought that patients would also be more likely to choose a shorter and less expensive treatment than RCT and therefore be more inclined to save teeth rather than extract them. Root canal treatment often involves extensive restoration of the tooth and possible subsequent laboratory made crown which adds additional costs to the therapy. Moreover, RCT and crown restorations frequently require more than one visit, which further increases the indirect costs and time spent.

**Table 1:** Prevalence of different pulp status in endodontic patients

**Pulp status**	**Frequency**	**Percent (%)**
**Normal**	6	1.6
**Reversible Pulpitis**	10	2.6
**Irreversible Pulpitis (IP)**	232	60.4
**Necrosis**	136	35.4
**Total**	**384**	**100**

According to the proposed agreed measures, the discussion panel concluded that CEM/VPT was more easily accessible, as the results for availability and cost were favorable.

**Impact**

The records (including radiographs) of 385 patients that attended the Endodontic Department of Shahid Beheshti Dental School in 2010 for RCT were evaluated. The number of teeth diagnosed with irreversible pulpitis or necrosis that required RCT was recorded. A total of 203 (52.9%) were maxillary and 181 (47.1%) were mandibular teeth. The majority of cases were molars (42.4 %), followed by premolars (38.5%), while anterior teeth and canines consisted of only 19% of total treated teeth. The judges disagreed on 7 cases (1.8% of total) which were excluded from the analysis.

Sixty percent of treated teeth were diagnosed with irreversible pulpitis and 35.4% with necrosis (Table 1). The prevalence of irreversible pulpitis was 60.7%, 68.2% and 43.8% in molars, premolars and anterior teeth, respectively. Premolars had the greatest diagnosis of irreversible pulpitis (Table 2); the difference was statistically significant (*P*>0.001). Only 29.8% of teeth diagnosed with irreversible pulpitis had a radiographic apical lesion, whereas 52.2% of necrotic teeth were diagnosed with chronic periapical periodontitis. The percentage of teeth with pulp necrosis compared to irreversible pulpitis in the three dental categories were significantly different (*P*=0.001) based on chi-square statistical test. The presence of periapical lesion in necrotic teeth versus those with irreversibly inflamed pulps were statistically different (*P*<0.001).

## Discussion

This is the first study in dentistry that employed HTA to evaluate a new biotechnology. CEM/VPT is a treatment alternative for management of irreversible pulpitis; the long-term efficacy and effectiveness of this new biotechnology has been recently investigated. The first phase of this study showed that based on short-term (post operative pain), intermediate- (6-month) and long-term (1- and 2-year) outcomes, this new biotechnology is successful even when patient factors are considered [18, 19, 25]. The qualitative safety assessment of VPT/CEM also showed that this is a reasonable treatment option in irreversible pulpitis when compared with RCT as the standard treatment. In fact, VPT/CEM can be more beneficial treatment, with good safety margins. Retreatment may be possible in the few failed cases as two year results show no evidence of root canal obliteration.

**Table 2:** Prevalence of irreversible pulpitis or necrosis

**Tooth**	**Irreversible pulpitis **		**Necrosis**	
	**n**	**%**	**n**	**%**
**1**	10	38.5	15	57.7
**2**	11	40.7	14	51.9
**3**	11	55.9	9	45.0
**4**	29	65.9	14	31.8
**5**	72	69.2	26	25.0
**6**	81	60.0	51	37.8
**7**	18	64.3	7	25.3

The many mishaps that may occur during RCT, such as iatrogenic perforation, patency at apical terminus and extrusion of root fillings, reduce the longevity of root treated teeth [32]. Moreover, there are strict radiographic and clinical criteria, such as Strindberg, to indicate good prognoses, and some general practitioners are not sufficiently skilled to attain this standard in molar teeth. Many root canal treatments would be termed failures radiographically. A recent meta-analysis showed that 36% of endodontically treated teeth have periradicular radiolucencies and 78% were reported to be technically inadequate [33].

A novel study has illustrated that the longevity of teeth treated with RCT, significantly increased when the mesial or distal proximal walls were maintained and when the tooth in question was not a molar. Therefore, in molar teeth a conservative cavity preparation and CEM/VPT instead of RCT may indeed increase their life span as pulp vitality and tooth proprioception is maintained, and less tissue is destroyed [34]. When the survival efficacy of the two interventions is compared, two year follow-up in root canal treated teeth is a good indicator of long-term success [7, 35, 36]. The results of recent trials showed that 6-month success rates of CEM/VPT was similar to the one- and two-year rates, however in the RCT arm, as time passed success rate of the remaining root treated teeth increased [18, 19]. That is, when root treated teeth are successful after 2 years, their likelihood of further success increases.

Cytotoxicity, genotoxicity and carcinogenicity tests have revealed that CEM is a biocompatible material similar to MTA [21, 31, 34, 37]. One study compared CEM with IRM and demonstrated that it was significantly more biocompatible [29] and others showed good bio-compatible clinical/histological responses when the material was used as a pulp cap or root-end filling material [30, 38-45].

CEM/VPT is more easily applicable compared to the more complex RCT, which is sometimes inadequately done [33]. CEM/VPT is also less costly and requires less specialist apparatus and materials, and therefore may have huge social and economical implications on oral health and patients’ quality of life in both developing and developed nations.

This study relied on the 2 year clinical outcomes of the randomized clinical trials; several studies have shown that 2 year outcome assessment is a sufficient prediction for the success or failure of the endodontic treatment [7, 36]. VPT/CEM may actually be a suitable treatment option for patients suffering from painful irreversible pulpitis as it is a quick solution that reduces pain more effectively than RCT. Also, it involves biomaterials with no filing or toxic irrigation. Additionally, radiography is not compulsory but advisable in this treatment for diagnosis and follow-up. This means that CEM/VPT has easier accessibility than RCT; however, the inability to retreat these teeth in cases of failure has not yet been reported.

The impact of CEM/VPT on Iranian oral health is significant when the percentage of patients suffering from irreversible pulpitis presenting to Shahid Beheshti Dental School is considered (60%); a value much higher than patients with necrotic pulps. Considering that in the last 10 years values of DMFT in a sample of 506 teenage students in Iran was relatively high (DMFT=1.8) [46], pulpotomy could greatly assist oral health in Iran.

The availability of the new biotechnology was judged to be far better due to less equipment, and training required. The overall results including the organizational measures and safety analyses can help to initiate adequate training and policy making in primary healthcare centers in Iran. It must be noted that training/education may incur an initial cost; however, the overall lower expected costs, this treatment modality will be more feasible than RCT in communities with limited funds.

Based on the results of present HTA, our suggestions are: *i)* incorporate this into undergraduate curriculum and into continuing professional education for qualified dentist; *ii)* increasing patient awareness towards prevention and pulpotomy instead of extraction in primary healthcare centers; and *iii)* incorporate the VPT/CEM treatment in dental insurance.

The similarities between Iran and other developing countries may also have social implications on their oral health policies and services [47]. 

## Conclusion

There was high-quality and long-term evidence from multicenter randomized clinical trials to support the use of VPT/CEM new biotechnology instead of RCT for patients suffering from *irreversible pulpitis*. Data relating to pain relief effect, radiographic outcomes, safety, costs, availability, accessibility and impact of VPT/CEM biotechnology, demonstrated superiority of VPT/CEM over RCT. We can conclude that VPT with a bio-regenerative material can be recommended for general clinical practice worldwide.
